# Geographic Variation in Pediatric Cancer Incidence — United States, 2003–2014

**DOI:** 10.15585/mmwr.mm6725a2

**Published:** 2018-06-29

**Authors:** David A. Siegel, Jun Li, S. Jane Henley, Reda J. Wilson, Natasha Buchanan Lunsford, Eric Tai, Elizabeth A. Van Dyne

**Affiliations:** ^1^Epidemic Intelligence Service, CDC; ^2^Division of Cancer Prevention and Control, National Center for Chronic Disease Prevention and Health Promotion, CDC.

Approximately 15,000 persons aged <20 years receive a cancer diagnosis each year in the United States (*1*). National surveillance data could provide understanding of geographic variation in occurrence of new cases to guide public health planning and investigation (*2*,*3*). Past research on pediatric cancer incidence described differences by U.S. Census region but did not provide state-level estimates (*4*). To adequately describe geographic variation in cancer incidence among persons aged <20 years in the United States, CDC analyzed data from United States Cancer Statistics (USCS) during 2003–2014 and identified 171,432 cases of pediatric cancer during this period (incidence = 173.7 cases per 1 million persons). The cancer types with the highest incidence rates were leukemias (45.7), brain tumors (30.9), and lymphomas (26.2). By U.S. Census region, pediatric cancer incidence was highest in the Northeast (188.0) and lowest in the South (168.0), whereas by state (including the District of Columbia [DC]), rates were highest in New Hampshire, DC, and New Jersey. Among non-Hispanic whites (whites) and non-Hispanic blacks (blacks), pediatric cancer incidence was highest in the Northeast, and the highest rates among Hispanics were in the South. The highest rates of leukemia were in the West, and the highest rates of lymphoma and brain tumors were in the Northeast. State-based differences in pediatric cancer incidence could guide interventions related to accessing care (e.g., in states with large distances to pediatric oncology centers), clinical trial enrollment, and state or regional studies designed to further explore variations in cancer incidence.

USCS includes incidence data from CDC’s National Program of Cancer Registries (NPCR) and the National Cancer Institute’s Surveillance, Epidemiology, and End Results (SEER) program (*1*). Data on new cases of cancer diagnosed during 2003–2014 were obtained from population-based cancer registries affiliated with NPCR and SEER programs in all U.S. states and DC. This study included incidence data for all registries that met USCS publication criteria* during 2003–2014, which represented >99% of the U.S. population, excluding data only from Nevada, which did not meet criteria in 2011. This report includes all cases of malignant^†^ cancer diagnosed among persons aged <20 years; it includes first primary cases only and excludes recurrent cases. Diagnosis histology and primary site were grouped according to the *International Classification of Childhood Cancer* (ICCC).^§^

Pediatric cancer rates were expressed per 1 million persons and were age-adjusted to the 2000 U.S. standard population.^¶^ Rates were estimated by sex, age group, race/ethnicity, state, U.S. Census region,** county-level economic status, county-level rural/urban classification, and ICCC group.

During 2003–2014, CDC identified 171,432 new cases of pediatric cancer (Table 1). Overall incidence was 173.7 cases per 1 million population. The cancer types with the highest incidence rates were leukemias (45.7 per 1 million), brain tumors (30.9), and lymphomas (26.2). Rates were higher in males (181.5) than in females (165.5) and in persons aged 0–4 years (228.9) and 15–19 years (213.3) than in persons aged 5–9 years (122.6) and 10–14 years (133.0). Among all racial/ethnic groups, the highest incidence rate was among whites (184.4), and the lowest was among blacks (133.3).

**TABLE 1:** Age-adjusted incidence rate* of cancer^†^ among persons aged <20 years, by U.S. Census region^§^ — United States,^¶^ 2003–2014

U.S. Census region										
Total			Northeast		Midwest		South		West	
Characteristic	No.	Rate (95% CI)	No.	Rate (95% CI)	No.	Rate (95% CI)	No.	Rate (95% CI)	No.	Rate (95% CI)
**Overall**	**171,432**	**173.7 (172.9–174.5)**	**31,893**	**188.0 (185.9–190.0)**	**37,702**	**172.9 (171.1–174.6)**	**61,998**	**168.0 (166.7–169.3)**	**39,839**	**172.9 (171.2–174.6)**
**Sex**										
Male	**91,667**	**181.5 (180.3–182.7)**	16,860	194.5 (191.6–197.5)	20,228	180.3 (178.8–182.8)	33,045	175.1 (173.3–177.0)	21,534	182.3 (179.9–184.8)
Female	**79,765**	**165.5 (164.3–166.6)**	15,033	181.1 (178.2–184.0)	17,474	164.3 (161.6–166.5)	28,953	160.6 (158.7–162.4)	18,305	163.0 (160.7–165.4)
**Age group (yrs)**										
0–4	**54,419**	**228.9 (227.0–230.8)**	9,467	242.7 (237.9–247.7)	12,001	227.0 (228.3–230.6)	20,161	222.7 (219.7–225.8)	12,790	226.1 (222.2–230.0)
5–9	**29,181**	**122.6 (121.2–124.1)**	5,161	128.7 (125.2–132.3)	6,323	121.2 (116.7–124.6)	10,862	121.4 (119.1–123.7)	6,835	123.2 (120.3–126.1)
10–14	**33,042**	**133.0 (131.5–134.4)**	6,256	145.1 (141.5–148.7)	7,128	131.5 (126.0–134.0)	12,042	130.4 (128.1–132.7)	7,616	131.9 (128.9–134.8)
15–19	**54,790**	**213.3 (211.5–215.1)**	11,009	238.5 (234.0–243.0)	12,250	211.5 (210.0–215.5)	18,933	200.5 (197.7–203.4)	12,598	213.5 (209.8–217.3)
Race/Ethnicity**										
White	**103,650**	**184.4 (183.3–185.5)**	21,580	200.8 (198.1–203.5)	28,309	183.3 (177.7–185.9)	34,798	178.9 (177.0–180.8)	18,963	184.9 (182.3–187.5)
Black	**20,188**	**133.3 (131.5–135.2)**	3,402	143.6 (138.8–148.5)	3,894	131.5 (125.4–135.6)	11,194	131.9 (129.5–134.4)	1,698	132.7 (126.4–139.1)
Hispanic	**36,197**	**168.9 (167.2–170.7)**	4,758	170.0 (165.2–175.0)	3,473	167.2 (153.5–170.2)	13,250	175.5 (172.5–178.5)	14,716	165.6 (162.9–168.3)
AI/AN	**1,507**	**147.6 (140.2–155.2)**	53	93.1 (69.7–121.9)	262	140.2 (118.9–155.2)	450	143.7 (130.7–157.6)	742	162.3 (150.8–174.5)
API	**7,089**	**144.6 (141.2–148.0)**	1,488	151.8 (144.2–159.8)	937	141.2 (133.6–148.0)	1,402	127.7 (121.1–134.6)	3,262	150.4 (145.3–155.7)
**County-level economic status by percentile**††										
≤25%	**19,536**	**165.7 (163.4–168.0)**	1,848	173.7 (165.9–181.9)	2,888	163.4 (162.3–168.7)	9,902	164.6 (161.3–167.8)	4,898	163.9 (159.3–168.5)
25–75%	**98,385**	**171.3 (170.2–172.4)**	15,032	182.2 (179.3–185.1)	21,073	170.2 (167.2–172.8)	38,515	167.8 (166.2–169.5)	23,765	172.1 (169.9–174.3)
≥75%	**48,268**	**181.8 (180.2–183.4)**	14,996	196.1 (193.0–199.3)	8,894	180.2 (175.8–183.3)	13,252	171.7 (168.8–174.7)	11,126	178.5 (175.2–181.9)
**County-level rural/urban continuum**††										
Metropolitan population ≥1 million	**93,181**	**177.1 (176.0–178.3)**	21,451	189.2 (186.6–191.7)	15,634	176.0 (171.5–178.0)	31,810	172.0 (170.2–173.9)	24,286	175.9 (173.6–178.1)
Metropolitan population 250,000 to <1 million	**35,919**	**171.1 (169.4–172.9)**	6,283	184.7 (180.2–189.4)	6,290	169.4 (169.1–172.7)	14,186	164.3 (161.6–167.0)	9,160	172.0 (168.5–175.6)
Metropolitan population <250,000	**14,349**	**165.7 (163.0–168.4)**	1,556	183.3 (174.2–192.7)	3,958	163.0 (161.0–168.4)	5,721	162.2 (158.0–166.5)	3,114	164.0 (158.3–169.8)
Nonmetropolitan counties	**22,962**	**167.2 (165.0–169.3)**	2,586	188.8 (181.5–196.3)	6,982	165.0 (165.3–169.3)	10,173	163.0 (159.9–166.2)	3,221	160.8 (155.3–166.4)

**Sources:** CDC’s National Program of Cancer Registries; National Cancer Institute’s Surveillance, Epidemiology, and End Results Program.

**Abbreviations:** AI/AN = American Indian/Alaska Native; API = Asian/Pacific Islander; CI = confidence interval.

* Rates are per 1 million persons and age-adjusted to the 2000 U.S. standard population.

^†^ Cases included all malignant cancers (with behavior code = 3) as grouped by the *International Classification of Childhood Cancer*.

*^§^ Northeast:* Connecticut, Maine, Massachusetts, New Hampshire, New Jersey, New York, Pennsylvania, Rhode Island, and Vermont. *Midwest:* Illinois, Indiana, Iowa, Kansas, Michigan, Minnesota, Missouri, Nebraska, North Dakota, Ohio, South Dakota, and Wisconsin. *South:* Alabama, Arkansas, Delaware, District of Columbia, Florida, Georgia, Kentucky, Louisiana, Maryland, Mississippi, North Carolina, Oklahoma, South Carolina, Tennessee, Texas, Virginia, and West Virginia. *West:* Alaska, Arizona, California, Colorado, Hawaii, Idaho, Montana, Nevada, New Mexico, Oregon, Utah, Washington, and Wyoming.

^¶^ Incidence data are compiled from cancer registries that meet the data quality criteria for all years 2003–2014 (covering >99% of the U.S. population). Nevada is excluded. Registry-specific data quality information is available at https://www.cdc.gov/cancer/npcr/uscs/pdf/uscs-2014-technical-notes.pdf. Characteristic values with other, missing, or blank results are not included in this table.

** White, black, AI/AN, and API persons are non-Hispanic. Hispanic persons might be of any race. Counts exclude unspecified or unknown race/ethnicity.

^††^ Excludes Kansas, Minnesota, and Nevada.

Rates were highest in the Northeast U.S. Census region, followed by the Midwest, the West, and the South. Rates were highest in the Northeast across all age groups and among whites and blacks. Among Hispanics, rates were highest in the South. Pediatric cancer incidence rates were highest in the 25% of counties with the highest economic status and were higher in metropolitan areas with populations ≥1 million than in nonmetropolitan areas.

By state, pediatric cancer incidence rates ranged from 145.2–205.5 per 1 million. Rates were highest in New Hampshire (205.5), DC (194.0), and New Jersey (192.3) and lowest in South Carolina (149.3) and Mississippi (145.2) (Table 2). Incidence among whites ranged from 157.0 in Montana to 255.2 in Hawaii; among blacks, from 105.8 in Rhode Island to 161.3 in Nebraska; and among Hispanics, from 75.0 in Hawaii to 191.8 in Florida.^††^ Although incidence rates were highest among children aged 0–4 years overall, in some states (e.g., New Jersey, New York, and Illinois), the highest rates were among persons aged 15–19 years (Supplementary Table 1, https://stacks.cdc.gov/view/cdc/53585).

**TABLE 2:** Age-adjusted incidence rate* of cancer^†^ among persons aged <20 years, by state, overall and by race/ethnicity — United States,^§^ 2003–2014

	Total		Race/Ethnicity^¶^									
			White		Black		Hispanic		AI/AN		API	
State**	No.	Rate (95% CI)	No.	Rate (95% CI)	No.	Rate (95% CI)	No.	Rate (95% CI)	No.	Rate (95% CI)	No.	Rate (95% CI)
**Northeast**												
Connecticut	**2,060**	**185.8 (177.8–194.0)**	1,399	194.8 (184.7–205.4)	199	144.6 (125.2–166.3)	361	176.8 (159.0–196.1)	—^††^	—^††^	63	133.1 (102.2–170.5)
Maine	**725**	**190.5 (176.9–205.0)**	685	194.8 (180.4–210.0)	—^††^	—^††^	—^††^	—^††^	—^††^	—^††^	—^††^	—^††^
Massachusetts	**3,584**	**181.5 (175.6–187.5)**	—^§§^	—^§§^	—^§§^	—^§§^	—^§§^	—^§§^	—^§§^	—^§§^	—^§§^	—^§§^
New Hampshire	**816**	**205.5 (191.6–220.2)**	746	207.6 (192.9–223.2)	—^††^	—^††^	31	177.8 (120.6–252.5)	—^††^	—^††^	18	157.1 (92.6–249.7)
New Jersey	**5,308**	**192.3 (187.1–197.5)**	3,168	211.8 (204.4–219.3)	633	148.6 (137.2–160.6)	1,043	175.2 (164.7–186.2)	—^§§^	—^§§^	345	145.7 (130.7–162.0)
New York	**11,378**	**190.0 (186.5–193.5)**	6,679	209.3 (204.3–214.4)	1,538	147.9 (140.6–155.5)	2,290	175.9 (168.7–183.2)	—^§§^	—^§§^	701	164.5 (152.5–177.1)
Pennsylvania	**7,167**	**186.6 (182.3–191.0)**	—^§§^	—^§§^	—^§§^	—^§§^	494	150.6 (137.6–164.6)	—^§§^	—^§§^	—^§§^	—^§§^
Rhode Island	**547**	**170.0 (156.0–185.0)**	429	196.3 (177.9–216.0)	28	105.8 (70.2–153.0)	59	96.8 (73.7–124.9)	—^††^	—^††^	—^††^	—^††^
Vermont	**308**	**164.2 (146.2–183.9)**	299	171.1 (152.0–191.9)	—^††^	—^††^	—^††^	—^††^	—^††^	—^††^	—^††^	—^††^
**Midwest**												
Illinois	**7,227**	**171.8 (167.9–175.8)**	4,320	183.9 (178.4–189.4)	934	124.4 (116.5–132.7)	1,548	171.2 (162.8–180.0)	—^§§^	—^§§^	273	146.7 (129.7–165.2)
Indiana	**3,691**	**171.5 (166.0–177.2)**	2,957	178.4 (172.0–185.0)	336	127.6 (114.4–142.1)	296	160.7 (142.7–180.4)	—^††^	—^††^	55	139.2 (104.7–181.3)
Iowa	**1,762**	**178.6 (170.4–187.2)**	1,508	181.2 (172.1–190.6)	60	115.7 (88.2–149.1)	130	166.2 (138.6–197.8)	—^††^	—^††^	30	140.0 (94.3–200.1)
Kansas	**1,713**	**177.0 (168.8–185.6)**	—^§§^	—^§§^	—^§§^	—^§§^	254	172.8 (152.0–195.7)	—^§§^	—^§§^	—^§§^	—^§§^
Michigan	**5,786**	**178.9 (174.3–183.6)**	4,339	188.1 (182.6–193.8)	826	140.5 (131.1–150.4)	296	135.8 (120.7–152.3)	34	127.1 (87.8–178.1)	116	122.3 (101.1–146.8)
Minnesota	**3,109**	**179.9 (173.6–186.3)**	2,420	181.4 (174.3–188.8)	177	122.8 (105.2–142.4)	203	162.6 (140.6–187.0)	46	159.1 (116.4–212.2)	159	162.2 (137.9–189.5)
Missouri	**3,120**	**163.1 (157.4–168.9)**	2,481	168.9 (162.3–175.6)	400	135.8 (122.8–149.8)	139	137.2 (115.0–162.3)	—^††^	—^††^	44	116.5 (84.6–156.5)
Nebraska	**1,133**	**183.2 (172.7–194.2)**	868	184.9 (172.8–197.7)	69	161.3 (125.3–204.2)	142	165.8 (139.2–196.0)	—^††^	—^††^	20	151.2 (92.2–233.7)
North Dakota	**341**	**158.7 (142.3–176.6)**	295	163.4 (145.2–183.2)	—^††^	—^††^	—^††^	—^††^	33	174.0 (119.6–244.7)	—^††^	—^††^
Ohio	**6,225**	**168.3 (164.1–172.5)**	4,999	175.6 (170.8–180.6)	751	124.5 (115.8–133.7)	206	122.2 (105.9–140.3)	—^††^	—^††^	106	147.5 (120.7–178.6)
South Dakota	**413**	**150.3 (136.1–165.5)**	347	162.4 (145.8–180.5)	—^††^	—^††^	—^††^	—^††^	49	126.9 (93.8–167.8)	—^††^	—^††^
Wisconsin	**3,182**	**175.6 (169.5–181.8)**	2,525	181.9 (174.8–189.1)	220	125.1 (109.1–142.7)	247	154.7 (135.7–175.4)	41	181.8 (130.3–246.7)	92	150.1 (120.9–184.1)
**South**												
Alabama	**2,377**	**157.0 (150.7–163.4)**	1,600	172.2 (163.8–180.8)	619	129.4 (119.4–140.1)	102	124.4 (100.7–152.0)	—^††^	—^††^	25	133.2 (86.1–196.8)
Arkansas	**1,523**	**161.7 (153.7–170.1)**	—^§§^	—^§§^	—^§§^	—^§§^	—^§§^	—^§§^	—^§§^	—^§§^	—^§§^	—^§§^
Delaware	**504**	**180.9 (165.5–197.5)**	—^§§^	—^§§^	—^§§^	—^§§^	—^§§^	—^§§^	—^§§^	—^§§^	—^§§^	—^§§^
District of Columbia	**306**	**194.0 (172.6–217.3)**	77	215.2 (165.9–274.7)	152	152.0 (128.7–178.2)	28	159.2 (104.6–231.4)	—^††^	—^††^	—^††^	—^††^
Florida	**9,160**	**169.9 (166.4–173.4)**	4,625	174.8 (169.8–179.9)	1,526	130.9 (124.4–137.6)	2,714	191.8 (184.7–199.2)	—^††^	—^††^	165	111.9 (95.5–130.4)
Georgia	**5,291**	**161.9 (157.6–166.3)**	2,884	177.1 (170.7–183.6)	1,556	136.2 (129.5–143.2)	634	166.9 (153.8–180.7)	—^††^	—^††^	159	144.2 (122.6–168.4)
Kentucky	**2,377**	**174.4 (167.4–181.5)**	—^§§^	—^§§^	—^§§^	—^§§^	—^§§^	—^§§^	—^§§^	—^§§^	—^§§^	—^§§^
Louisiana	**2,378**	**156.9 (150.7–163.4)**	1,453	177.7 (168.7–187.1)	753	127.1 (118.2–136.5)	113	164.2 (134.8–198.0)	—^††^	—^††^	42	173.9 (125.3–235.1)
Maryland	**2,942**	**160.0 (154.2–165.9)**	1,664	179.7 (171.2–188.6)	773	125.1 (116.4–134.3)	286	156.0 (138.1–175.4)	—^††^	—^††^	99	95.1 (77.2–115.8)
Mississippi	**1,476**	**145.2 (137.9–152.8)**	860	166.0 (155.1–177.5)	548	121.7 (111.7–132.4)	45	138.5 (100.2–186.3)	—^††^	—^††^	—^††^	—^††^
North Carolina	**4,834**	**161.6 (157.1–166.2)**	3,052	175.2 (169.0–181.5)	991	129.3 (121.4–137.7)	560	155.6 (142.6–169.4)	38	88.7 (62.8–121.8)	111	138.6 (113.9–167.1)
Oklahoma	**2,082**	**168.3 (161.1–175.6)**	1,273	166.1 (157.0–175.4)	170	131.0 (112.0–152.2)	276	168.9 (149.2–190.4)	296	194.1 (172.6–217.5)	36	142.5 (99.8–197.4)
South Carolina	**2,162**	**149.3 (143.1–155.8)**	1,370	164.7 (156.1–173.6)	600	122.2 (112.6–132.4)	149	154.4 (130.0–182.0)	—^††^	—^††^	24	114.2 (73.1–170.0)
Tennessee	**3,411**	**172.1 (166.4–178.0)**	2,500	180.4 (173.4–187.6)	614	144.5 (133.3–156.4)	211	160.4 (138.7–184.4)	—^††^	—^††^	48	142.2 (104.7–188.6)
Texas	**16,368**	**183.2 (180.4–186.0)**	6,598	200.7 (195.8–205.6)	1,571	140.0 (133.1–147.1)	7,503	179.7 (175.6–183.8)	47	162.0 (118.8–216.0)	431	134.0 (121.6–147.3)
Virginia	**3,899**	**156.4 (151.5–161.4)**	2,553	169.2 (162.7–175.9)	710	124.1 (115.1–133.6)	355	139.1 (124.8–154.5)	—^††^	—^††^	175	118.2 (101.3–137.1)
West Virginia	**908**	**172.0 (160.9–183.5)**	855	175.4 (163.8–187.5)	28	110.2 (73.1–159.3)	—^††^	—^††^	—^††^	—^††^	—^††^	—^††^
**West**												
Alaska	**424**	**169.4 (153.6–186.3)**	232	158.0 (138.3–179.7)	—^††^	—^††^	25	138.7 (89.5–204.3)	115	217.2 (179.3–260.7)	40	232.0 (165.7–316.0)
Arizona	**3,590**	**168.8 (163.3–174.4)**	1,683	176.1 (167.8–184.7)	130	122.4 (102.2–145.3)	1,454	164.4 (156.1–173.1)	199	164.2 (142.1–188.7)	79	132.7 (105.0–165.5)
California	**21,725**	**173.2 (170.9–175.6)**	7,505	189.9 (185.6–194.2)	1,184	137.9 (130.1–146.0)	10,525	170.1 (166.9–173.4)	101	138.7 (112.8–168.8)	2,187	148.3 (142.1–154.6)
Colorado	**2,767**	**171.3 (165.0–177.8)**	1,754	175.6 (167.4–184.0)	103	121.7 (99.3–147.6)	762	162.4 (151.1–174.5)	20	153.2 (93.2–237.6)	88	171.8
Hawaii	**652**	**160.1 (148.0–172.9)**	134	255.2 (213.7–302.4)	—^††^	—^††^	46	75.0 (54.3–101.0)	—^††^	—^††^	439	155.6
Idaho	**941**	**170.0 (159.3–181.3)**	789	178.3 (166.0–191.2)	—^††^	—^††^	121	136.5 (113.1–163.3)	—^††^	—^††^	—^††^	—^††^
Montana	**488**	**160.2 (146.2–175.0)**	398	157.0 (141.9–173.2)	—^††^	—^††^	24	162.8 (104.0–242.7)	56	182.4 (137.7–237.0)	—^††^	—^††^
New Mexico	**1,077**	**157.0 (147.7–166.6)**	393	198.7 (179.5–219.4)	20	126.9 (77.5–196.1)	539	139.7 (128.2–152.0)	101	131.0 (106.7–159.2)	16	186.7 (106.6–303.7)
Oregon	**2,114**	**182.6 (174.9–190.6)**	1,591	192.1 (182.7–201.8)	40	111.6 (79.7–152.0)	343	155.1 (139.0–172.6)	27	134.5 (88.5–196.4)	81	146.1 (116.0–181.6)
Utah	**1,984**	**178.3 (170.5–186.4)**	1,596	182.2 (173.3–191.3)	23	130.1 (82.1–195.9)	309	180.9 (161.1–202.5)	—^††^	—^††^	40	120.5 (86.0–164.0)
Washington	**3,797**	**180.7 (175.0–186.5)**	2,656	189.8 (182.6–197.2)	163	135.8 (115.8–158.4)	542	146.9 (134.6–159.9)	83	200.1 (159.3–248.2)	276	158.1 (140.0–177.9)
Wyoming	280	156.8 (139.0–176.3)	232	159.1 (139.3–181.0)	—^††^	—^††^	26	118.1 (76.8–173.4)	—^††^	—^††^	—^††^	—^††^

**Sources:** CDC’s National Program of Cancer Registries; National Cancer Institute’s Surveillance, Epidemiology, and End Results Program.

**Abbreviations:** AI/AN = American Indian/Alaska Native; API = Asian/Pacific Islander; CI = confidence interval.

* Rates are per 1 million persons and age-adjusted to the 2000 U.S. standard population.

^†^ Cases included all malignant cancers (with behavior code = 3) as grouped by the *International Classification of Childhood Cancer*.

^§^ Incidence data are compiled from cancer registries that meet the data quality criteria for all years 2003–2014 (covering >99% of the U.S. population). Nevada is excluded. Registry-specific data quality information is available at https://www.cdc.gov/cancer/npcr/uscs/pdf/uscs-2014-technical-notes.pdf.

^¶^ White, black, AI/AN, and API are non-Hispanic. Hispanic persons might be of any race. Counts exclude unspecified or unknown race/ethnicity; the counts in the total column may not equal the sum of the individual race/ethnicity columns.

** States are grouped by U.S. Census region.

^††^ Case counts <16 are suppressed.

^§§^ Race/ethnicity data was suppressed for states that elected to be excluded from race/ethnicity analysis.

Pediatric cancer incidence rates varied by state within each cancer type (Figure). Incidence rates were highest in the West for leukemias, myeloproliferative diseases, and myelodysplastic diseases (ICCC group I) and in the Northeast for lymphomas and reticuloendothelial neoplasms (group II) and central nervous system cancers (group III). Rates were also highest in the Northeast for neuroblastoma, retinoblastoma, bone tumors, soft tissue sarcomas, and thyroid cancer (Supplementary Table 2, https://stacks.cdc.gov/view/cdc/53586). Renal cancer rates were highest in the Northeast and South; hepatic tumor rates were highest in the Northeast and West. Germ cell tumor rates were highest in the West (Supplementary Table 2, https://stacks.cdc.gov/view/cdc/53586).

**FIGURE:**
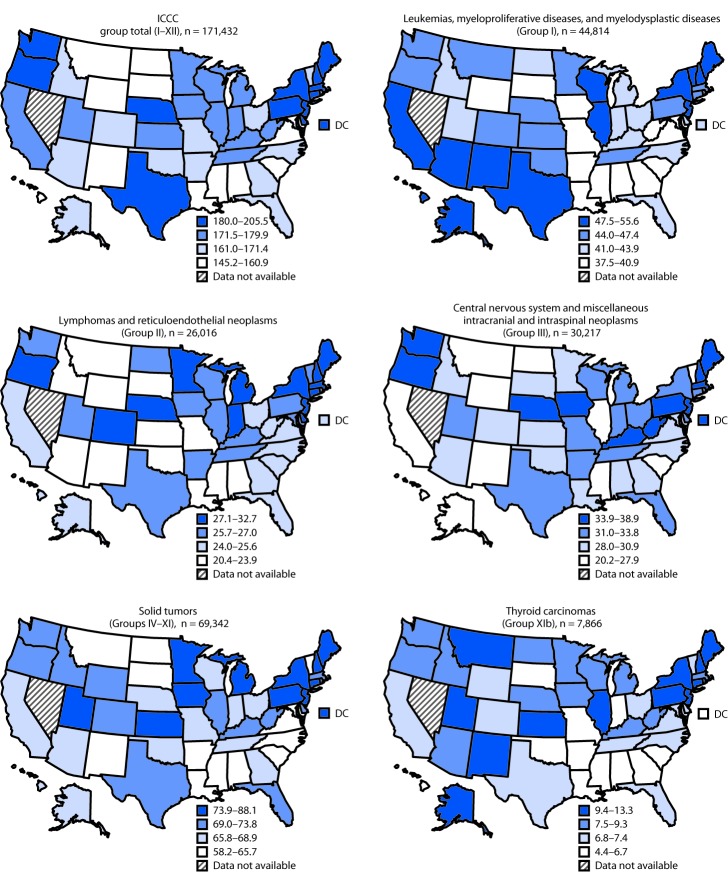
Age-adjusted incidence* of cancer^†^ among persons aged <20 years, by U.S. state and ICCC type — United States,^§^ 2003–2014^¶^ **Sources:** CDC’s National Program of Cancer Registries; National Cancer Institute’s Surveillance, Epidemiology, and End Results Program. **Abbreviation:** ICCC = International Classification of Childhood Cancer. * Rates are per 1 million persons and age-adjusted to the 2000 U.S. standard population. ^†^ Cases included all malignant cancers (with behavior code = 3) as grouped by the ICCC. ^§^ Solid tumors (Groups IV–XI) include neuroblastoma and other peripheral nervous cell tumors, retinoblastoma, renal tumors, hepatic tumors, malignant bone tumors, soft tissue and other extraosseous sarcomas, germ cell and trophoblastic tumors and neoplasms of gonads, and other malignant epithelial neoplasms and melanomas. The ICCC group total map includes 258 cases not classified by ICCC. ^¶^ Incidence data are compiled from cancer registries that meet the data quality criteria for all years 2003–2014 (covering >99% of the U.S. population). Nevada is excluded. Registry-specific data quality information is available at https://www.cdc.gov/cancer/npcr/uscs/pdf/uscs-2014-technical-notes.pdf.

## Discussion

This study used recent data with greater population coverage than past studies (*4*,*5*) to document geographic variation in pediatric cancer incidence rates by sex, age, type, and race/ethnicity. Consistent with past reports (*1**,**4**,**5*), pediatric cancer rates were highest in males, persons aged 0–4 years and 15–19 years, whites, and the Northeast U.S. Census region. Rates were highest in metropolitan areas with populations ≥1 million; state-based rates were highest in New Hampshire, DC, and New Jersey.

A strength of this report is the use of extensive population-based surveillance data (>99% coverage^§§^), which permits a detailed description of state-based cancer incidence variation. Geographic variation in rates might account for differences in results from previous studies that were based on different populations such as state data (*2*,*3*), SEER registries (which cover 9%–28% of the U.S. population),^¶¶^ or other large data sets (*6*). A 2016 study specific to Delaware assessed pediatric cancer incidence by demographic group and ZIP Code; the study commented on local environmental exposures and possible incidence disparities based upon sex, age, race/ethnicity, geographic location, and economic status (*2*). USCS data provide states with a standardized way to gauge whether local pediatric cancer incidence rates differ relative to other states and might prompt states to conduct investigations similar to the one performed in Delaware.

Geographic variation in pediatric cancer incidence might be influenced by several factors.*** First, variation in childhood cancer incidence might be related to differences in exposures to carcinogenic chemicals (e.g., air pollution, secondhand smoke, food, or drinking water) or radiation (*7*). Second, genetic variation in certain populations (e.g., prevalence of cancer predisposition genes) (*2*,*4*,*5*) might contribute to geographic differences in cancer incidence. Third, the rates of certain cancer types might vary by race/ethnicity. For example, Hispanic children have the highest rate of the most common type of leukemia, pediatric acute lymphoblastic leukemia, and states with a higher proportion of Hispanics might have higher rates of acute lymphoblastic leukemia (*8*). Fourth, incidence of some types of cancer (e.g., thyroid carcinoma) might be related to enhanced detection and access to care, which can vary by geographic location (*5*,*9*).

In addition, geographic variation might be affected by age, economic status, or rural/urban classification (*4*,*8*,*10*). Similar to the findings from this report, recent data detailing adult cancers also indicate that the highest cancer incidence rates are in the Northeast (*10*). Rates of cancer types mostly affecting adults also varied by rural/urban status; some of these differences in adults might be related to factors such as obesity or smoking (*10*), which might or might not also explain rural/urban variation in pediatric cancer.

The findings in this report are subject to at least three limitations. First, Nevada was excluded because data for 2011 did not meet quality criteria, which limits the representativeness of the findings. Second, differences in diagnosis and cancer reporting among states might contribute to variation in cancer incidence rates (*8*). For example, states that were early adopters of electronic pathology reporting might report increased rates because of increased case ascertainment compared with other states. Finally, misrepresentation of race and ethnicity might exist; rate numerators might underestimate American Indians, Alaska Natives, and Hispanics, which could artificially lower rates among these groups; and U.S. Census populations used in rate denominators might undercount children and Hispanics, which could artificially increase rates in these populations (*8*).^†††^

Knowledge of pediatric cancer incidence variation by state and cancer type can prompt local and state cancer registries to evaluate reporting and diagnostic standards. Understanding geographic variation in incidence rates can help cancer control planners and clinicians address obstacles in access to care, which is especially relevant to states with large distances to pediatric oncology centers (*3*). Because 5-year pediatric cancer survival is >80%, and most cancer survivors require close monitoring by specialists throughout life (*5*), state-specific data by cancer type and patient age might help public health planners address ongoing chronic care needs. In addition, state-specific data by cancer type and patient age might help clinical trial organizers predict patient accrual. Finally, health care practitioners and researchers can use these data to guide investigations related to causes of pediatric cancer incidence variation (*2*,*3*). Continued surveillance will be needed to further validate findings and track geographic incidence patterns over time.

**Summary**

**What is already known about this topic?**

Past research on nationwide pediatric cancer incidence described differences by U.S. Census region but did not provide state-level estimates.

**What is added by this report?**

During 2003–2014, the pediatric cancer rate was highest in the Northeast, lowest in the South, and highest in metropolitan areas with populations ≥1 million and counties in the top 25% economic status. Incidence rates by state ranged from 145 to 206 per million and were highest in New Hampshire, the District of Columbia, and New Jersey. The highest rate of leukemia was in the West; the highest rates of lymphoma and brain cancer were in the Northeast.

**What are the implications for public health practice?**

Knowledge of these geographic differences in childhood cancer incidence can be used to enhance provider awareness, treatment capacity, survivorship care, and cancer surveillance.

## Footnotes

* Cancer registries’ incidence data met the following five USCS criteria: 1) ≤5% of cases ascertained solely on the basis of death certificate; 2) ≤3% of cases missing information on sex; 3) ≤3% of cases missing information on age; 4) ≤5% of cases missing information on race; and 5) ≥97% of registry’s records passed a set of single-field and interfield computerized edits that test the validity and logic of data components. https://nccd.cdc.gov/uscs/.

† Used behavior code = 3. https://seer.cancer.gov/behavrecode/.

§ https://seer.cancer.gov/iccc/iccc-who2008.html and https://onlinelibrary.wiley.com/doi/full/10.1002/cncr.20910. The ICCC applies the rules and nomenclature of the *International Classification of Diseases for Oncology, Third Edition*: http://codes.iarc.fr/.

¶ Population estimates incorporate bridged single-race estimates derived from the original multiple race categories in the 2010 U.S. Census. https://seer.cancer.gov/popdata.

** https://www.census.gov/geo/reference/gtc/gtc_census_divreg.html.

†† State-specific rate ranges by race/ethnicity do not include data suppressed for states that elected to be excluded from race/ethnicity analysis.

§§ https://www.cdc.gov/cancer/npcr/uscs/pdf/uscs-2014-technical-notes.pdf.

¶¶ https://seer.cancer.gov/registries/data.html.

*** https://www.cdc.gov/cancer/npcr/uscs/data/00_guidance_include.htm.

†††
https://www.cdc.gov/cancer/npcr/uscs/technical_notes/interpreting/race.htm .
